# Supplementary material for: Resolving the full spectrum of human genome variation using Linked-Reads
Source: Genome Res. 2019 Apr;29(4):635–45. doi: 10.1101/gr.234443.118 (PMC6442396; doi:10.1101/gr.234443.118)
Supplement: Supplemental Material [file supp_gr.234443.118_Supplemental_longranger-2.2.zip › longranger-2.2.tar.bz2!/lr2.2-public/lib/stan/cmdstan/stan/doc/api/html/check__simplex_8hpp_source.html]

Stan: src/stan/math/error\_handling/matrix/check\_simplex.hpp Source File


|  |  |
| --- | --- |
|  | Stan  2.5.0  probability, sampling & optimization |


- Main Page
- Related Pages
- Namespaces
- Classes
- Files

- File List
- File Members

All Classes Namespaces Files Functions Variables Typedefs Enumerations Enumerator Friends Macros Pages

- src
- stan
- math
- error\_handling
- matrix

check\_simplex.hpp

Go to the documentation of this file.

1 #ifndef \_\_STAN\_\_MATH\_\_ERROR\_HANDLING\_\_MATRIX\_\_CHECK\_SIMPLEX\_HPP\_\_

2 #define \_\_STAN\_\_MATH\_\_ERROR\_HANDLING\_\_MATRIX\_\_CHECK\_SIMPLEX\_HPP\_\_

3

4 #include <sstream>

5 #include <stan/math/error\_handling/dom\_err.hpp>

6 #include <stan/math/error\_handling/matrix/constraint\_tolerance.hpp>

7 #include <stan/math/matrix/Eigen.hpp>

8 #include <stan/math/matrix/meta/index\_type.hpp>

9 #include <stan/meta/traits.hpp>

10

11

12 namespace stan {

13

14  namespace math {

15

31  template <typename T\_prob, typename T\_result>

32  bool check\_simplex(const char\* function,

33  const Eigen::Matrix<T\_prob,Eigen::Dynamic,1>& theta,

34  const char\* name,

35  T\_result\* result) {

36  using Eigen::Dynamic;

37  using Eigen::Matrix;

38  typedef typename index\_type<Matrix<T\_prob,Dynamic,1> >::type size\_t;

39

40  if (theta.size() == 0) {

41  std::stringstream msg;

42  msg << " is not a valid simplex. "

43  << "length(" << name << ") = %1%";

44  std::string tmp(msg.str());

45  return dom\_err(function,0,name,

46  tmp.c\_str(),"",

47  result);

48  }

49  if (!(fabs(1.0 - theta.sum()) <= CONSTRAINT\_TOLERANCE)) {

50  std::stringstream msg;

51  T\_prob sum = theta.sum();

52  msg << " is not a valid simplex.";

53  msg.precision(10);

54  msg << " sum(" << name << ") = " << sum

55  << ", but should be %1%";

56  std::string tmp(msg.str());

57  return dom\_err(function,1.0,name,

58  tmp.c\_str(),"",

59  result);

60  }

61  for (size\_t n = 0; n < theta.size(); n++) {

62  if (!(theta[n] >= 0)) {

63  std::ostringstream stream;

64  stream << " is not a valid simplex. "

65  << name << "[" << n + stan::error\_index::value << "]"

66  << " = %1%, but should be greater than or equal to 0";

67  std::string tmp(stream.str());

68  return dom\_err(function,theta[n],name,

69  tmp.c\_str(),"",

70  result);

71  }

72  }

73  return true;

74  }

75

76  }

77 }

78 #endif

dom\_err.hpp

stan::agrad::fabs

fvar< T > fabs(const fvar< T > &x)

**Definition:** fabs.hpp:18

stan::math::dom\_err

bool dom\_err(const char \*function, const T &y, const char \*name, const char \*error\_msg, const T\_msg error\_msg2, T\_result \*result)

**Definition:** dom\_err.hpp:36

stan::math::check\_simplex

bool check\_simplex(const char \*function, const Eigen::Matrix< T\_prob, Eigen::Dynamic, 1 > &theta, const char \*name, T\_result \*result)

Return true if the specified vector is simplex.

**Definition:** check\_simplex.hpp:32

constraint\_tolerance.hpp

traits.hpp

stan::math::CONSTRAINT\_TOLERANCE

const double CONSTRAINT\_TOLERANCE

The tolerance for checking arithmetic bounds In rank and in simplexes.

**Definition:** constraint\_tolerance.hpp:11

stan::math::index\_type

Primary template class for the metaprogram to compute the index type of a container.

**Definition:** index\_type.hpp:21

stan::error\_index::value

**Definition:** traits.hpp:20

index\_type.hpp

Eigen.hpp

stan::math::sum

double sum(std::vector< double > &x)

**Definition:** sum.hpp:10


---

[ Stan Home Page ]

*© 2011–2014,
Stan Development Team.*
